# In Silico Design and Computational Elucidation of Hypothetical Resveratrol–Curcumin Hybrids as Potential Cancer Pathway Modulators

**DOI:** 10.3390/ph18101473

**Published:** 2025-09-30

**Authors:** Nil Sazlı, Deniz Karataş

**Affiliations:** Bioengineering Department, Manisa Celal Bayar University, Yunusemre, Manisa 45140, Turkey; 231208005@ogr.cbu.edu.tr

**Keywords:** de novo curcumin-resveratrol hybrids, in silico drug design, cancer signaling pathways, molecular modelling, structural and pharmacokinetic properties

## Abstract

**Background/Objectives:** Cancer progression is characterized by the suppression of apoptosis, activation of metastatic processes, and dysregulation of cell proliferation. The proper functioning of these mechanisms relies on critical signaling pathways, including Phosphoinositide 3-kinase/Protein kinase B/mammalian Target of Rapamycin (PI3K/Akt/mTOR), Mitogen-Activated Protein Kinase (MAPK), and Signal Transducer and Activator of Transcription 3 (STAT3). Although curcumin and resveratrol exhibit anticancer properties and affect these pathways, their pharmacokinetic limitations, including poor bioavailability and low solubility, restrict their clinical application. The aim of our study was to evaluate the synergistic anticancer potential of curcumin and resveratrol through hybrid molecules rationally designed from these compounds to mitigate their pharmacokinetic limitations. Furthermore, we analyzed the multi-target anticancer effects of these hybrids on the AKT serine/threonine kinase 1 (AKT1), MAPK, and STAT3 pathways using in silico molecular modeling approaches. **Methods:** Three hybrid molecules, including a long-chain (ELRC-LC) and a short-chain (ELRC-SC) hybrid, an ester-linked hybrid, and an ether-linked hybrid (EtLRC), were designed using the Avogadro software (v1.2.0), and their geometry optimization was carried out using Density Functional Theory (DFT). The electronic properties of the structures were characterized through Frontier Molecular Orbital (FMO), Molecular Electrostatic Potential (MEP), and Fourier Transform Infrared (FTIR) analyses. The binding energies of the hybrid molecules, curcumin, resveratrol, their analogs, and the reference inhibitor were calculated against the AKT1, MAPK, and STAT3 receptors using molecular docking. The stabilities of the best-fitting complexes were evaluated through 100 ns molecular dynamics (MD) simulations, and their binding free energies were estimated using the Molecular Mechanics/Poisson–Boltzmann Surface Area (MM/PBSA) method. **Results:** DFT analyses demonstrated stable electronic characteristics for the hybrids. Molecular docking analyses revealed that the hybrids exhibited stronger binding compared to curcumin and resveratrol. The binding energy of −11.4 kcal/mol obtained for the ELRC-LC hybrid against AKT1 was particularly remarkable. Analysis of 100 ns MD simulations confirmed the conformational stability of the hybrids. **Conclusions:** Hybrid molecules have been shown to exert multi-target mechanisms of action on the AKT1, MAPK, and STAT3 pathways, and to represent potential anticancer candidates capable of overcoming pharmacokinetic limitations. Our in silico-based study provides data that will guide future in vitro and in vivo studies. These rationally designed hybrid molecules, owing to their receptor affinity, may serve as de novo hybrid inhibitors.

## 1. Introduction

Cancer cases, characterized by multilayered biological disorders such as uncontrolled cell proliferation, activation of invasion–metastasis processes, and deactivation of apoptosis mechanisms, have been increasing in recent years [1]. Accordingly, although many traditional methods such as chemotherapy and radiotherapy are used for cancer treatment, they exhibit side effects and possess the potential to develop resistance in cancer cells [2,3,4]. In recent years, the most prominent plant-derived compound in cancer research has been the polyphenolic molecule curcumin, obtained from *Curcuma longa* [5,6]. However, the chemical structure of curcumin reduces the clinical potential of this natural compound. This has led to the synthesis of new curcumin analogs that exhibit greater target specificity and stronger anticancer activity than curcumin [5,7]. A literature study employing in silico methods demonstrated that curcumin analogs exhibit significantly better binding affinity to target receptors such as Nuclear Factor kappa-light-chain-enhancer of activated B cells (NF-κB) and Epidermal Growth Factor Receptor (EGFR), which are associated with cancer, compared to curcumin [5]. In addition, the analysis of the biological activities of the analogs, using in vitro methodologies such as the resazurin cell viability assay and reactive oxygen species (ROS) measurement in lung cancer cells (A549), reported that the analogs have a different mechanism of action [8]. Conjugates, molecular hybrids, and monocarbonyl derivatives are particularly important for enhancing the therapeutic effectiveness of curcumin. Replacement of the β-diketone structure of curcumin with a monocarbonyl structure indicates that curcumin analogs possess strong antioxidant activity, whereas monocarbonyl hybrids of curcumin containing a *1,2,3-triazole* ring enhance its water solubility [9]. A separate literature study on the curcumin–triazole hybrid reported that the hybrid exhibited antiproliferative activity in MCF-7 and MDA-MB-231 human breast cancer cell lines, as well as in 4T1 mouse breast cancer cells, with an IC_50_ of 70 µM for pure curcumin, which decreased to 6–10 µM for the hybrid. This finding suggests that the hybrid induces apoptosis by modulating intracellular signaling proteins [10]. Similarly, the study by Flint et al. demonstrated significant selectivity in breast cancer cell lines for the 47c derivative of asymmetric diarylidenone (“dienone”) curcumin analogs, with IC_50_ values. These findings indicate that curcumin derivatives approximate standard chemotherapeutic agents in terms of tumor selectivity and efficacy. Furthermore, Absorption, Distribution, Metabolism, Excretion, and Toxicity (ADMET) analyses by Flint et al. revealed that certain curcumin analogs exhibit significantly higher oral absorption than curcumin and also display profiles such as reduced blood–brain barrier permeability [11]. These studies on curcumin, a naturally occurring compound, demonstrate the significant role of curcumin in cancer biology [10,11]. In contrast, another polyphenolic compound is resveratrol. Resveratrol, present in various plants such as grapes, is considered a highly valuable compound for cancer chemoprevention. This has been shown to be effective in both the initiation and progression of carcinogenesis, and to modulate signaling pathways regulating angiogenesis and metastasis. Numerous studies have reported that resveratrol induces apoptosis and reduces invasive and metastatic capacity in cancers such as lung, prostate, breast, and colon [12,13]. This compound, in particular, induces cell cycle arrest in the G1 and S phases and reduces the levels of anti-apoptotic proteins. An in vitro study on resveratrol reported that it activates the tumor suppressor protein p53 and triggers caspase-3, thereby initiating programmed cell death [14]. Additionally, resveratrol was found to dose-dependently attenuate invasion and cell viability, and to suppress the expression of the oncogenic factor FBJ murine osteosarcoma viral oncogene homolog (FOS) and the matrix metalloproteinase-9 (MMP-9). These findings suggest that resveratrol also targets tumor cell invasion and metastatic progression [15]. Resveratrol’s effects on cancer therapy are undeniable; however, similar to curcumin, it has certain limitations, including poor water solubility, low bioavailability, and the requirement for high effective doses. In various studies, resveratrol has shown an IC_50_ of approximately highlighting the need to investigate new analogs to enhance its therapeutic efficacy [16,17,18]. For example, substituting a methoxy group in the chemical structure of resveratrol increased its lipophilicity, thereby facilitating its passage through the cell membrane. This modification conferred significantly improved pharmacokinetic properties [19]. Another study revealed new resveratrol analogs with enhanced antitumor activity, attributed to double-bond modifications and skeletal changes in their structure. Additionally, there is literature on the hybridization of curcumin and resveratrol, as well as different molecular scaffolds [20]. In the study conducted by Simon et al. curcumin–curcumin hybrid structures provide low IC_50_ values and high selectivity in breast cancer cell lines such as MCF-7 and MDA-MB-231 [21]. Similarly, studies have shown that resveratrol can form self-hybrid structures, leading to structural O-methylation and, consequently, increased lipophilicity, thereby effectively stimulating apoptotic mechanisms [11]. As evidenced by the reviewed studies, structural optimization of curcumin and resveratrol, their analogs, and related hybrid molecules underscores the value of this approach [11]. These two natural compounds, whether in their native form, as novel analogs, or as hybrid derivatives exhibit promising anticancer potential against a range of cancer types. Therefore, molecular-level combination of these two compounds, which share similar pharmacokinetic limitations such as low absorption, provide complementary mechanisms of action. Moreover, it enhances cell-targeting capacity and biological activity. Studies exploring hybrid compounds represent an important strategy for overcoming tumor cell resistance mechanisms in cancer treatment. Recent studies have further reported that resveratrol and curcumin, acting as a synergistic combined agent, are effective against cancer through multiple mechanisms [22]. It is hypothesized that they may exert a synergistic effect when used in combination. For example, a study by Du et al. demonstrated that these two molecules produced a synergistic effect by inducing cell death beyond expected levels in cell culture experiments, with a combination index (CI) < 1.0. Biological examination of this synergistic interaction revealed increased endoplasmic reticulum stress in cancer cells, activation of autophagic cell death pathways, significant elevation of ROS levels, and suppression of the Phosphoinositide 3-kinase/Protein kinase B/mammalian Target of Rapamycin (PI3K/Akt/mTOR) pathway. In a separate study, this combination was found to disrupt the resistance mechanisms of epithelial ovarian cancer cells resistant to cisplatin treatment. This effect was attributed to the synergy between curcumin and resveratrol, which inhibited the PI3K/Akt/mTOR pathway [22]. A separate study reported that curcumin and resveratrol, when covalently bound, exert an antiproliferative effect on estrogen receptor-positive breast cancer cells. Notably, this compound inhibited specific phases of the cell cycle in MCF-7 cells and exhibited no toxicity in normal fibroblast cells [23]. In a study on colorectal cancer, the hybrid molecules were predicted to induce apoptosis by strongly binding to caspase-3/7, the tumor suppressor protein p53, and matrix metalloproteinase-7 (MMP-7) [24]. Studies employing molecular modeling methodologies have demonstrated that the curcumin–resveratrol hybrid compound drug-like properties due to its potential interactions with target proteins [25]. This molecular-level analysis of the cytotoxic effects of the hybrid compound in SW480 and SW620 cell lines [24,25]. Unlike the compounds targeted against colorectal cancer reported in the study by Moreno et al. [24], our study introduces distinct hybrid molecules designed through esterification and de-etherification methodologies. Whereas previous studies relied solely on docking, our work demonstrates the development of structural specificity by applying a comprehensive methodology to oncogenic proteins (AKT1, MAPK, STAT3) using DFT, molecular docking, molecular dynamics simulations, and MM/PBSA free energy calculations [25].

Based on the aforementioned studies, we anticipate that pharmacokinetic limitations—such as poor in vitro bioavailability, low water solubility, and rapid metabolism—can be addressed through de novo hybrid compounds generated by covalently combining resveratrol and curcumin via esterification and etherification reactions. In this context, two ester-linked resveratrol–curcumin hybrids (ELRC-LC, long-chain; ELRC-SC, short-chain) and one ether-linked resveratrol–curcumin hybrid (EtLRC), differing in side-chain length and degree of conjugation, were designed in silico. For all these reasons, our study suggests that the newly developed synergistic hybrids of resveratrol and curcumin may not only improve pharmacokinetic properties but also confer multi-target anticancer mechanisms. Molecular modeling analyses, including binding affinity, target protein specificity, and interaction sites, will systematically elucidate the cancer-associated biological pathways that contribute to multi-target anticancer potential. Our study offers a unique contribution to natural synergistic drug discovery by in silico modeling of molecular interaction networks. These hybrid molecules are a new generation of highly bioavailable synergistic anticancer natural agents capable of suppressing cancer cell proliferation, overcoming chemotherapy resistance, and exhibiting pharmaceutical suitability.

## 2. Results

### 2.1. DFT-Based Electronic Properties and Spectroscopic Analyses

**Figure 1:**
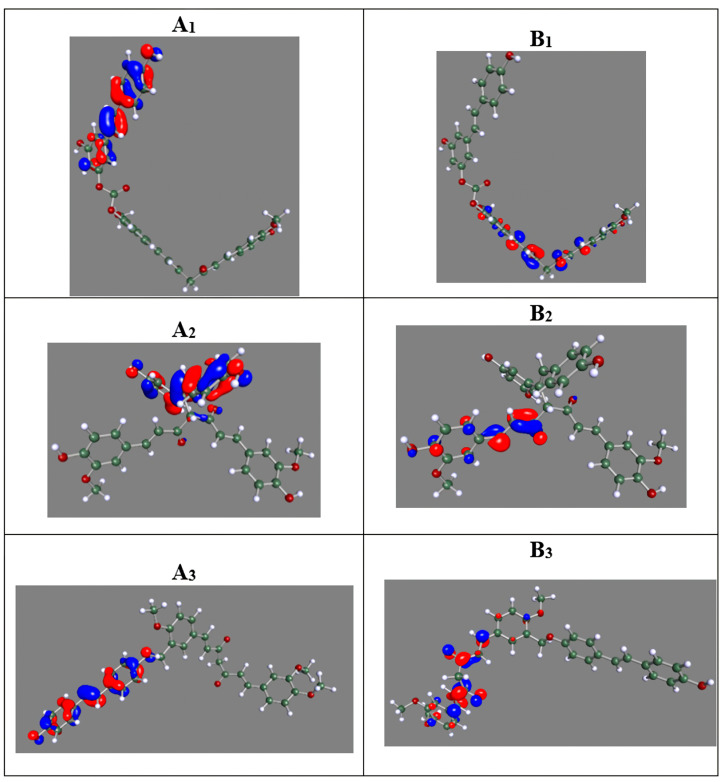
The frontier molecular orbital (FMO) distributions of the designed hybrids were visualized using the T-MoleX program. Panels (**A_1_**–**A_3_**) show the highest occupied molecular orbitals (HOMO) of the ELRC-LC, ELRC-SC, and EtLRC molecules, respectively, while panels (**B_1_**–**B_3_**) represent the lowest unoccupied molecular orbitals (LUMO) of the corresponding hybrids. The red and blue regions in the images indicate the different phases of the wave function in the orbital density maps. These phases denote the separation of electron-donor (HOMO) and electron-acceptor (LUMO) sites, reflecting the electronic properties associated with skeletal modifications of the ligands.

**Figure 2:**
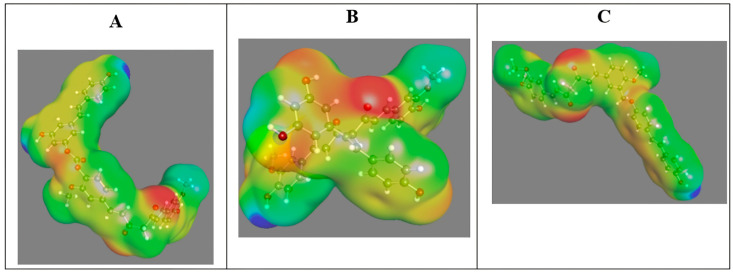
Molecular Electrostatic Potential (MEP) surface maps of the hybrid molecules are shown in the figure. (**A**) ELRC-LC, (**B**) ELRC-SC, and (**C**) EtLRC represent the hybrid ligands. MEPs are mapped on electron density isosurfaces (±0.07 e/au^3^), and the color scale, defined as electron-poor (blue) and electron-rich (red), provides information about the potential interaction sites of the ligands in the binding pockets of the receptors. The ligands in question have similar C=C bonds in their aliphatic chains and structurally similar electronic characteristics. However, the absence of conjugation results in the lack of distinct absorption bands in the spectra.

In our study, after designing ELRC-LC, ELRC-SC, and EtLRC hybrids, the most physicochemically stable conformations were optimized using Density Functional Theory (DFT) calculations. The frontier molecular orbital (HOMO–LUMO), molecular electrostatic potential (MEP) distributions, and Fourier Transform Infrared (FTIR) spectra of these optimized hybrids were computed. The HOMO–LUMO images, which illustrate the key electronic properties of the hybrid molecules, are presented in Figure 1 and Table 1, the MEP maps in Figure 2, and the FTIR vibrational peaks in Table 2. The HOMO–LUMO distributions of these hybrid molecules are distributed in distinct regions. As shown in Figure 1A_1_–A_3_, the HOMO is predominantly localized on the phenolic rings, whereas the LUMO is distributed along the extended aliphatic chains. In the ELRC-SC hybrid, the HOMO is mainly localized on the ester bridge, while the LUMO is concentrated on the phenolic terminal groups (Figure 1B_2_). In the EtLRC structure, the HOMO and LUMO overlap in the phenolic and ether bridge regions, respectively (Figure 1A_3_,B_3_). The numerical values of the HOMO–LUMO energy levels and the derivative reactivity parameters, including the HOMO–LUMO energy gap (ΔE), electronegativity (χ), hardness (η), softness (σ), chemical potential (μ), and electrophilicity index (ω), are presented in Table 1. According to our results, ELRC-SC shows the strongest chemical stability with the widest GAP value of 3.6154 eV. ELRC-LC demonstrates a relatively stable profile, with a GAP value of 3.2318 eV compared to the EtLRC hybrid. However, a striking finding is that EtLRC has the highest electrophilicity index, indicating its potential susceptibility to electrophilic attacks. In the MEP analyses shown in Figure 2, nucleophilic character is evident at the carbonyl oxygens of all hybrids, whereas electrophilic character is prominent at the aliphatic hydrogens. In the common FTIR findings of the curcumin- and resveratrol-based hybrid molecules presented in Table 2, phenolic C–O–C groups were observed for curcumin in the region of 1139–1271 cm^−1^, while C–O–C ester groups were observed for resveratrol. In the range of 1650–1750 cm^−1^, ester and conjugated carbonyl groups were observed, respectively. However, after hybridization, the aliphatic C–H stretching observed in experimental results appears in the 2920–2960 cm^−1^ region, whereas in the hybrids these values shift to around 3013–3014 cm^−1^. Similarly, aromatic C=C vibrations are reported in the 1600–1640 cm^−1^ range in the literature, while they shift to 1610–1650 cm^−1^ in the hybrid molecules. In the 3700–3800 cm^−1^ range, the O–H bands are observed to be preserved in the hybrids. Furthermore, although the C=O band is detected at 1752 cm^−1^ in resveratrol in experimental results, it is noteworthy that it shifts to the 1724 cm^−1^ region in the EtLRC hybrid.

### 2.2. Molecular Docking Results

**Figure 3:**
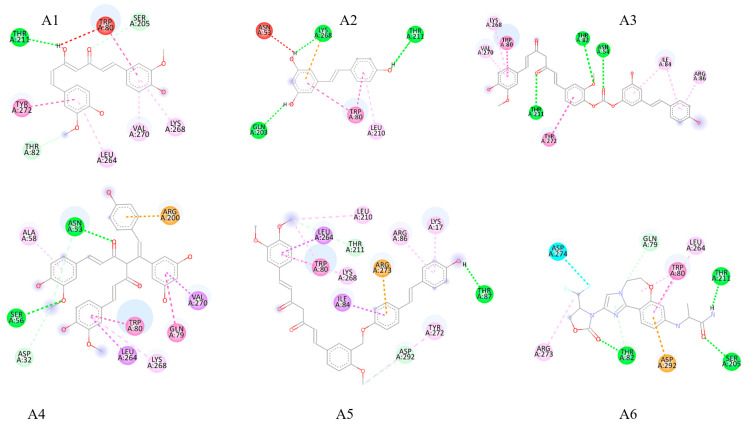

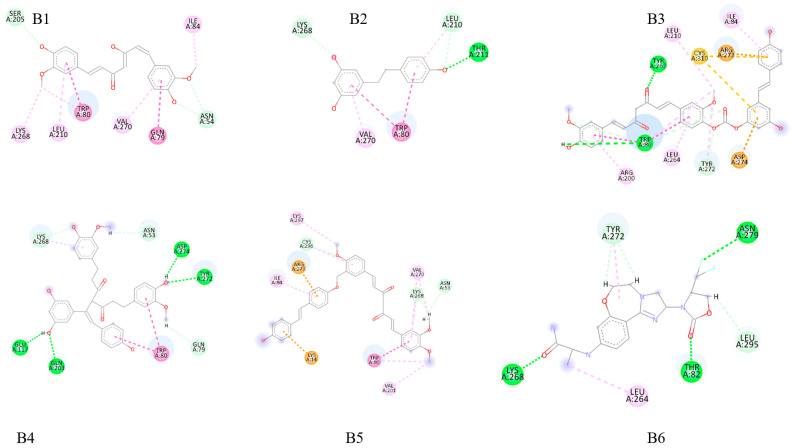

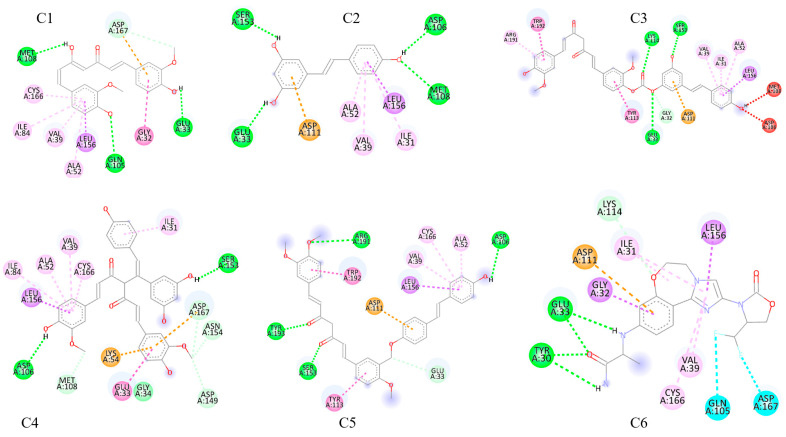

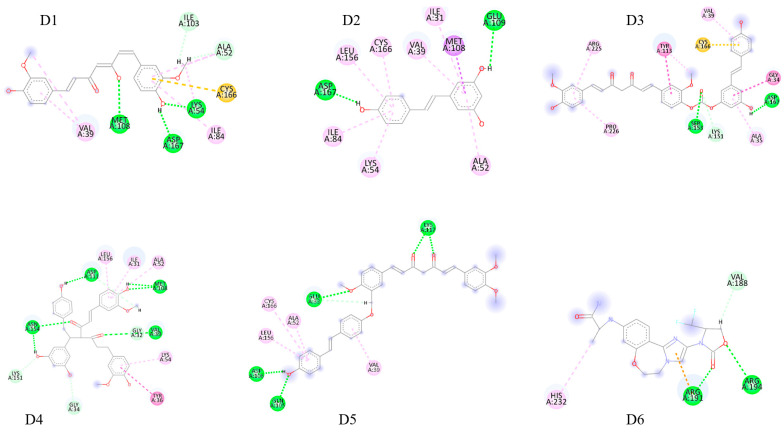

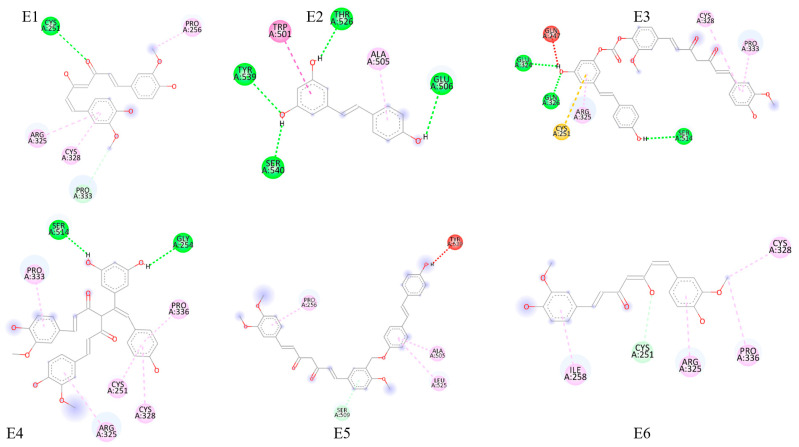

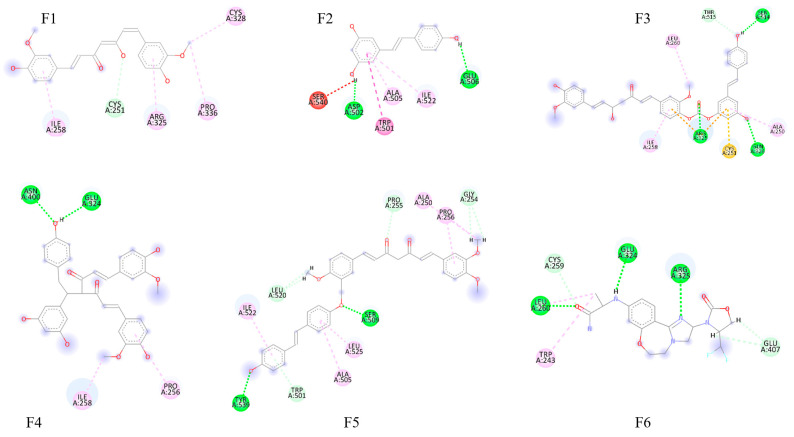
Interaction maps of curcumin, resveratrol, the hybrid ligands, and the reference inhibitor inavolisib on the AKT1, MAPK, and STAT3 target receptors. Panels (**A1**–**A6**) illustrate the interaction profiles of the AKT1 receptor with the ligands: (**A1**) curcumin, (**A2**) resveratrol, (**A3**) ELRC-LC, (**A4**) ELRC-SC, (**A5**) EtLRC, and (**A6**) inavolisib after docking. Panels (**B1**–**B6**) display the interactions of the same ligands with AKT1 in the last frame (100 ns) of the MD simulation. Panels (**C1**–**C6**) show the interactions of the MAPK receptor after docking, while panels (**D1**–**D6**) represent the interactions of the same receptor and ligands in the last frame of the MD simulation. Panels (**E1**–**E6**) illustrate the binding interactions of the ligands on STAT3 after docking, and panels (**F1**–**F6**) depict the binding profiles of the ligands on the STAT3 receptor after MD simulation. Conventional hydrogen bonds are represented by dashed green lines, alkyl and π-alkyl interactions by pink lines, carbon–hydrogen bonds by light green lines, π-sigma interactions by purple dashed lines, unfavorable acceptor–acceptor interactions by red dashed lines, and π–π T-shaped interactions by dark pink lines.

Table 3 presents the binding energies (kcal/mol) of curcumin, resveratrol, their analogs, the hybrid molecules, and the reference inhibitor inavolisib against receptors involved in the AKT serine/threonine kinase 1 (AKT1), Mitogen-Activated Protein Kinase (MAPK), and Mitogen-Activated Protein Kinase (STAT3) signaling pathways, calculated using molecular docking. Compared to the binding energies of the single curcumin and resveratrol complexes, the analogs and hybrid molecules exhibited stronger binding affinities with lower binding energy values. The results indicate that the ELRC-LC hybrid molecule exhibits a favorable binding profile, with binding energies of −11.4 kcal/mol on the AKT1 receptor, −9.6 kcal/mol on the MAPK receptor, and −5.7 kcal/mol on the STAT3 receptor. Similarly, the ELRC-SC hybrid shows binding energies of −9.5, −7.8, and −7.2 kcal/mol with the AKT1, MAPK, and STAT3 receptors, respectively, whereas the EtLRC hybrid exhibits binding energies of −10.8, −9.3, and −7.6 kcal/mol. ELRC-LC stands out due to its binding superiority toward AKT1, while EtLRC demonstrates a stable binding conformation with comparable binding values across all three receptors. Additionally, the binding energies of the reference inhibitor were −9.9, −8.1, and −7.2 kcal/mol on the AKT1, MAPK, and STAT3 receptors, respectively, indicating that the hybrid molecules achieved significantly better results than the reference inhibitor. The binding mechanisms of these hybrids, the individual natural polyphenols, and the reference inhibitor with the receptor active sites are illustrated in Figure 3. These binding maps reveal that the hybrids establish multiple contacts, exhibiting a broader range of binding interactions.

### 2.3. Molecular Dynamics (MD) Simulation

**Figure 4:**
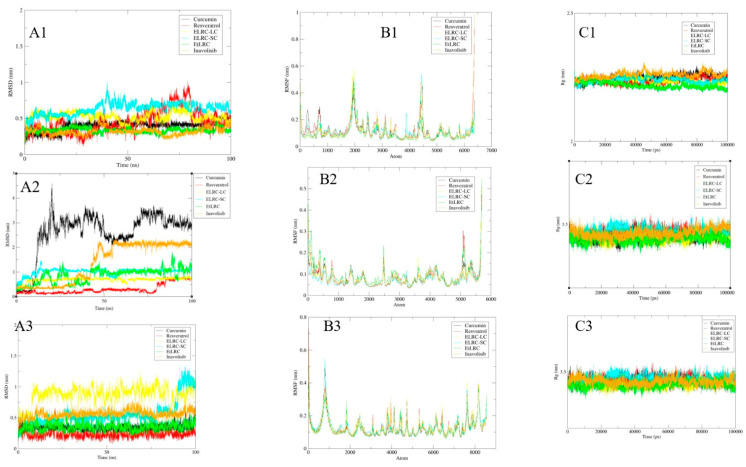
MD simulations of the AKT1, MAPK, and STAT3 receptors were performed with complexes of the natural polyphenols curcumin and resveratrol, the designed hybrid molecules (ELRC-LC, ELRC-SC, EtLRC), and the reference inhibitor inavolisib for 100 ns. Root Mean Square Deviation (RMSD) profiles of the receptor complexes ((**A1**) AKT1, (**A2**) MAPK, (**A3**) STAT3) were generated to measure conformational stability. Root Mean Square Fluctuation (RMSF) plots ((**B1**) AKT1, (**B2**) MAPK, (**B3**) STAT3) were prepared to analyze residue-based flexibility, and radius of gyration (Rg) analyses ((**C1**) AKT1, (**C2**) MAPK, (**C3**) STAT3) were performed to evaluate global compactness. All profiles were visualized using the QtGrace program. The black, red, yellow, cyan, green, and orange lines in the graphs represent curcumin, resveratrol, ELRC-LC, ELRC-SC, EtLRC, and inavolisib, respectively.

After docking, 100 ns molecular dynamics (MD) simulations were performed to determine the optimal conformations of the receptor–ligand complexes. The binding stability of the ligands on the target receptors was evaluated using RMSD analysis (Figure 4A1–A3). Curcumin and the natural polyphenol hybrids appear to reach equilibrium rapidly in the simulation on the AKT1 receptor. The EtLRC hybrid stands out with its very low RMSD values of approximately 0.3 nm, maintaining stability throughout the entire simulation. At the MAPK receptor, curcumin and the reference inhibitor exhibit higher RMSD values compared to the other ligands (Figure 4A2). The hybrid molecules exhibited lower RMSD values and less fluctuation within the range of 0–1 nm. Resveratrol, however, showed the lowest stability at approximately 0.3 nm. Notably, the ELRC-LC hybrid reached a plateau at around 0.8 nm, the ELRC-SC hybrid plateaued at 1.0 nm, and the EtLRC hybrid increased to 1.5 nm after 80 ns. On the STAT3 receptor, all hybrid molecules maintained RMSD values below 1 nm (Figure 4A3). The resveratrol complex exhibited a more stable profile in this case.

**Figure 5:**
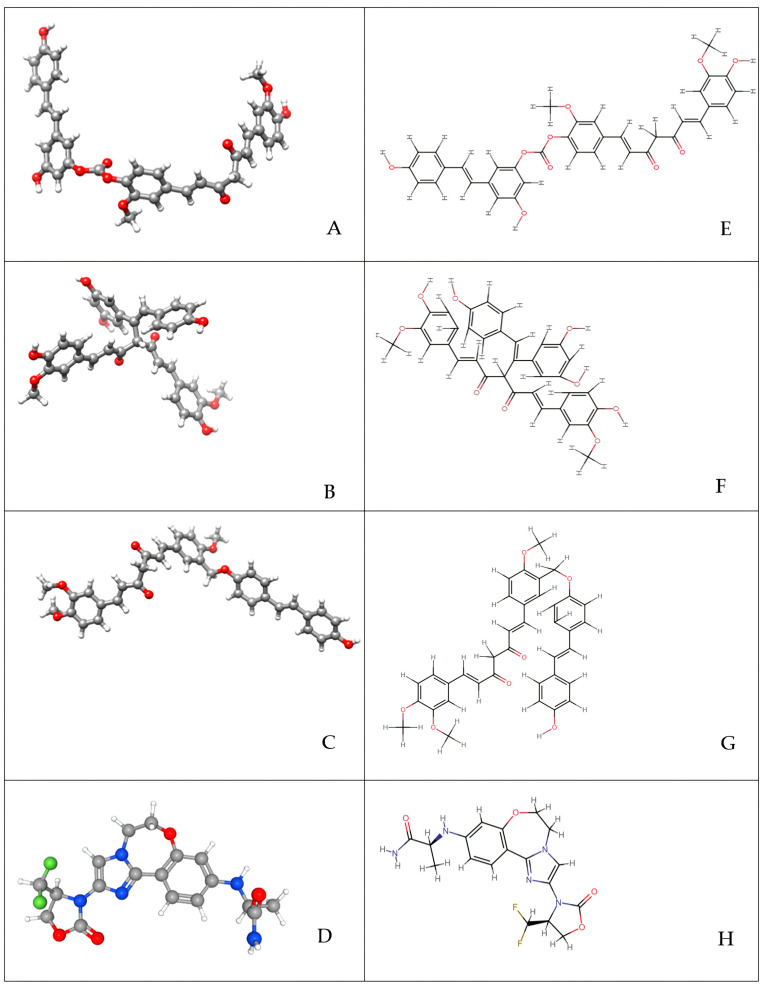
Figures of three different curcumin–resveratrol hybrid molecules designed within the scope of our study were visualized using Avogadro (v1.2.0) software. (**A**) It represents the long-chain ester-linked hybrid structure (ELRC-LC), which was designed by covalently binding the phenolic hydroxyl group of the resveratrol structure to a long alkyl-chain activated carboxylic acid derivative derived from the β-diketone region of curcumin. (**B**) It represents the short-chain ester-linked curcumin–resveratrol hybrid (ELRC-SC), which has a similar synthesis strategy as the ELRC-LC hybrid but has a shorter chain length. Finally, (**C**) represents the ether-linked curcumin–resveratrol hybrid (EtLRC) structure designed based on the principles of Williamson ether synthesis. The 3D chemical structure is presented in panel (**D**) to compare the chemical structure with the reference inhibitor, inavolisib. The 2D chemical structures of the hybrids are presented in panels (**E**–**H**), corresponding to the ELRC-LC, ELRC-SC, and EtLRC hybrids, inavolisib, respectively. In the 3D structures of all hybrid molecules, gray, red, white, blue, and green colors represent carbon (C), oxygen (O), hydrogen (H), nitrogen (N), and fluorine (F), respectively.

Findings regarding amino acid flexibility were obtained through RMSF analyses and are presented in Figure 5B1–B3. The peaks of the complexes were generally observed in the loop and terminal regions. The RMSF values of the hybrid molecules ranged between 0.1 and 0.5 nm, and the peaks appeared suppressed. In contrast, the resveratrol ligand exhibited pronounced fluctuations in certain loop regions, reflecting higher flexibility within the receptor. This suggests that resveratrol is less restrictive than the other ligands. The hybrid molecules, in particular, exhibit increased local rigidity compared to inavolisib. The compactness profiles of the receptor–ligand complexes were analyzed using the radius of gyration (R*g*), as shown in Figure 5C1–C3. On the AKT1 receptor, all ligands generally maintained compactness values between 2.0 and 2.25 nm (Figure 4C1). The stability of ELRC-LC and EtLRC at approximately 2.2 and 2.15 nm, respectively, suggests that the receptors maintain their structural integrity. ELRC-SC, on the other hand, exhibits a stable profile at 2.2 nm. The natural polyphenols displayed a compact structural profile similar to that of the hybrids, whereas the reference inhibitor exhibited a looser conformation with a higher Rg value. For the MAPK receptor, these values increased to approximately 3.5 nm (Figure 4C2). The EtLRC hybrid exhibited the narrowest fluctuation range at approximately 3.2 nm. ELRC-LC demonstrated greater compactness compared to ELRC-SC. Curcumin and resveratrol showed compactness with values around 3.4 nm, whereas the reference inhibitor displayed a more irregular profile on this receptor. On the STAT3 receptor, the ligands maintained stability between 3.2 and 3.5 nm. Although curcumin and resveratrol polyphenols exhibited broad fluctuations, they retained an overall compact structure. In contrast, the low-fluctuation plateaus of the ELRC-LC and EtLRC hybrids highlighted these two molecules with compactness profiles on this receptor.

### 2.4. Binding Free Energy Analysis Using Molecular Mechanics/Poisson–Boltzmann Surface Area (MM/PBSA)

The principal component values of the binding free energies of the receptor–ligand complexes in their best conformations, calculated using the Molecular Mechanics/Poisson–Boltzmann Surface Area (MM/PBSA) method, are presented in Table 4. The ΔG_bind_ values of curcumin, one of the natural polyphenols, in the last frame of the AKT1, MAPK, and STAT3 receptors were −13.39, −32.28, and −17.60 kcal/mol, respectively, whereas for resveratrol they were −12.46, −17.20, and −20.57 kcal/mol. The EtLRC hybrid molecule exhibited a ΔG_bind_ value of −26.50 kcal/mol on AKT1, with van der Waals and electrostatic contributions calculated as −75.42 and −29.64 kcal/mol, respectively. At the end of the simulation, the energy value showed a tolerable shift of −25.19 kcal/mol, indicating stability. The ELRC-LC and ELRC-SC hybrids exhibited favorable binding energy values of −22.11 and −22.95 kcal/mol, respectively. At the MAPK receptor, ELRC-SC exhibited a pronounced decrease to −36.29 kcal/mol, representing the strongest binding complex. At the STAT3 receptor, the EtLRC hybrid stood out with an energy of −35.40 kcal/mol, while values close to or even better than those of the reference inhibitor placed the hybrid molecules at the forefront in terms of stability and binding affinity.

## 3. Discussion

### 3.1. Density Functional Theory (DFT) Analysis

#### 3.1.1. Frontier Molecular Orbitals (FMO) Analysis

The HOMO and LUMO analyses presented in Figure 1 provide crucial insights into the electronic reactivity, molecular structure, and stability of the hybrid molecules. In the ELRC-LC hybrid, the HOMO density is predominantly localized on the phenolic rings, whereas the LUMO distribution is mainly concentrated in the long aliphatic chains. This spatial separation of orbitals highlights the electron-donating nature of the aromatic regions, which facilitates hydrogen bonding and π–π stacking interactions within the hybrid [26,27]. The hydrophobic tails of this structure represent the electron-acceptor regions and facilitate penetration into the apolar subpockets of the receptor [28]. In the ELRC-SC molecule, the HOMO orbital density is localized around the central ester bridge, whereas the LUMO is predominantly concentrated in the phenolic terminal groups. This hybrid structure exhibits electron-accepting capacity at the functional groups and electron-donating ability from the compact bridge region, thereby contributing to selective stabilization at the polar amino acid sites [29]. The narrower orbital distribution in ELRC-SC compared to ELRC-LC suggests a more localized electronic structure. This finding provides preliminary evidence that conformational stability can be achieved in molecular dynamics simulations [29,30]. EtLRC exhibits a distinct electronic profile compared to the other hybrids, with HOMO and LUMO overlapping in both the phenolic regions and the ether bridge. This hybrid displays ambifunctional electronic characteristics, functioning simultaneously as an electron donor and acceptor within the same structural domains. Such dual behavior facilitates the formation of water-mediated hydrogen bond networks and promotes cohesive binding in the polar regions of proteins [31]. Preliminary inferences suggest that the chemical structure of EtLRC significantly enhances the solvent sensitivity of its bidirectional electronic activity, which may account for the fluctuations observed in the RMSD plots (Figure 4A3).

In addition to the visual representations of the hybrids’ Frontier Molecular Orbital (FMO) analyses (Figure 1), the HOMO–LUMO energy gap (ΔE) and other reactivity parameters presented in Table 1 further support our findings. The ELRC-SC hybrid displays the widest GAP (3.6154 eV), indicating high chemical hardness and enhanced stability. The calculated η value of 1.8077 eV further confirms the consistency of these results. In comparison, ELRC-LC exhibits relatively greater stability than the other hybrid, EtLRC. However, the relatively narrow GAP value of EtLRC, 2.8272 eV, is attributed to the increased polarity and electron distribution provided by the ether bridge in its structure. This finding makes the hybrid more electrophilic [32]. The HOMO values of the hybrids range from −5.6607 to −5.1430 eV, and the LUMO values range from −2.3468 to −2.0453 eV, which is consistent with the localized analysis of the orbitals and quantitatively emphasizes that these molecules possess electron-acceptor and donor characteristics in different regions [27]. A study by Boulmokh et al. reported that curcumin and its derivatives exhibit HOMO orbital density in the phenolic rings, while the LUMO is localized in the side chains. This has been reported to enhance reactivity and provide curcumin molecules with a soft electrophilic character [26]. In the study conducted by Lima et al. on resveratrol, it was observed that the HOMO–LUMO distributions varied significantly depending on solvent polarity and influenced biological reactivity in a parallel manner [27]. In our study, the orbital separations of the designed hybrid molecules preserved the electronic character of individual curcumin and resveratrol molecules and positively influenced their binding adaptability through structural modifications, which is consistent with the literature findings.

#### 3.1.2. Molecular Electrostatic Potential (MEP) Analysis

Molecular Electrostatic Potential (MEP) maps are presented in Figure 1 to evaluate the electrostatic distributions of the designed hybrid molecules and to identify potential reactive sites within the binding pockets. The MEP map of ELRC-LC (Figure 1A) reveals neutral regions along the extended aliphatic chain, which enable van der Waals-based stabilization with the hydrophobic pockets of the receptor [33]. The intense red regions indicate the strong nucleophilic character of the carbonyl oxygens, whereas the blue regions highlight the electrophilic character of the aliphatic and phenolic hydrogens [34]. The MEP analysis of EtLRC indicates that it enhances binding stability and hydrogen-bond acceptor capacity through hydrophobic packing [28]. In the ELRC-SC hybrid (Figure 1B), the sharp color boundaries around the carbonyl and phenolic regions suggest a compact electronic distribution. The carbonyl oxygens act as nucleophilic centers; however, the short aliphatic chain imposes certain limitations on hydrophobic surface adaptation, thereby favoring localized hydrogen-bonding interactions [28,34]. This finding is consistent with the FMO results for EtLRC, demonstrating selective electrostatic interactions within the binding pocket. The ether oxygens of EtLRC, which display a distinct profile compared to the other hybrids, exhibit pronounced nucleophilicity in the red regions (Figure 2B). The polarity of the ether bridge facilitates the formation of solvent-mediated hydrogen bonds [33]. These findings provide insights into the possible causes of electrostatic fluctuations observed in analyses such as the RMSD during MD simulations [33,35]. They indicate that the ether bridge may contribute to binding interactions with the receptor, both directly and through solvent-mediated effects [36]. In general, the carbonyl oxygens of the hybrid molecules exhibit nucleophilic character, whereas the aliphatic and phenolic hydrogens display electrophilic character [37]. The hydrophobic adaptation of the ELRC-LC, the directional hydrogen-bond specificity of the ELRC-SC hybrid, and the solvent-mediated electrostatic flexibility of the EtLRC hybrid are quite remarkable. The MEP findings of the hybrids are consistent with the FMO findings, representing the partially electrophilic nature of the carbonyl groups. The findings of Wang et al. in their photoisomerization studies of resveratrol isomers, which revealed that the hydroxyl oxygens can act as nucleophilic and electrophilic centers, indicate that our study is consistent with the literature [38].

#### 3.1.3. FTIR (Fourier Transform Infrared) Spectroscopy Analysis

The Fourier Transform Infrared (FTIR) analysis results confirming the functional groups of the hybrid molecules designed in this study are presented in Table 2. Theoretical vibrational modes of the hybrids derived from the combination of curcumin and resveratrol molecules were compared with experimental data reported in the literature. The characteristic peak values of the hybrid molecules showed overall consistency with the literature findings. The C=O stretching vibrations detected at 1639 cm^−1^ in curcumin and at 1752 cm^−1^ in resveratrol were generally observed in the range of 1650–1748 cm^−1^ in the hybrid molecules [39,40]. These spectral shifts are within a tolerable range and confirm the consistency of our findings. The observed variations are attributed to the influence of hydrogen bonding and π-conjugation effects [41]. Furthermore, the presence of the carbonyl band in resveratrol indicates ester and ketonic functionalities within the hybrid structures [42]. The aromatic C=C stretching modes at ~1600–1650 cm^−1^ correspond to the stabilization of the phenyl ring systems of both curcumin and resveratrol, indicating the preservation of this structural integrity in the hybrids. Additionally, the C–O phenolic modes of curcumin and the C–O–C ester-related modes of resveratrol observed at peak values around 1000–1200 cm^−1^ confirm the contribution of phenolic hydroxyl and ester bonds to the hybrid structures [39,40]. Similarly, the C–H asymmetric stretching vibrations (CH_2_, CH_3_) associated with alkyl side-chain structures in the range of 2900–3016 cm^−1^ indicate the spectral activity of the hybrid molecules [39]. These FTIR spectral shifts indicated that the hybrid molecules retained their characteristic functional groups, demonstrating that structural integrity was maintained at the molecular level. While this finding does not provide direct evidence of biological stability, it is consistent with our results from other complementary DFT, molecular docking, MD simulation, and MM/PBSA analyses, suggesting that the ligands retain the functions necessary for binding to target receptors. Experimental peaks reported in the literature exhibit slight shifts in the hybrid structures (Table 2). While the aliphatic –CH_3_ peak is typically observed around 2922–2960 cm^−1^, it generally shifts toward the 3000–3019 cm^−1^ region in the hybrid models. The C=O band observed at 1752 cm^−1^ in the resveratrol polyphenol shifts to 1724 cm^−1^ in the EtLRC hybrid, attributed to the rearrangement of the conjugative effects of functional groups within the ether bridge. Comparable shifts caused by π-conjugation effects and hydrogen-bond rearrangements have also been documented in the literature [43]. O–H stretching bands were observed in the hybrid molecules in the range of 3740–3802 cm^−1^. The differences in intensity and width of these stretching bands represent strong hydrogen-bond networks. The presence of these bands in the range of 3174–3265 cm^−1^ in experimental findings is attributed to variations in hydrogen bonding caused by ambient polarity and in silico calculation conditions [44]. In summary, our findings suggest that these molecules contribute to stability in biological environments. Similar results emphasize the critical role of ester and ether bridges in hybrids, influencing the vibrational behavior of π-conjugation and hydrogen bonding [43]. Comparison with the literature indicates that curcumin and resveratrol polyphenols in the hybrids retain their essential functional groups and, in addition, exhibit structural modifications that enhance biological activity.

**Table 2:** Comparative FTIR vibrational frequencies of the designed hybrids with experimental peaks.

Prominent Theoretical Peaks (cm^−1^) *			Experimental Peak (cm^−1^)	Assignment
ELRC-LC	ELRC-SC	EtLRC		
1650, 1654, 1722, 1828	1650, 1656, 1730, 1748	1631, 1724	1639 (Curcumin) [39]; 1752 (Resveratrol) [40]	C=O stretching (ester; conjugated carbonyl)
1620, 1625, 1643, 1612, 1616, 1636	1616, 1619, 1627, 1637, 1635, 1648, 1653	1626, 1651, 1549, 1607, 1652	1639 (Curcumin) [39]; 1600–1640 (Resveratrol) [40]	C=C aromatic stretching (phenyl ring)
1139, 1219		1023, 1271	1079 (Curcumin) [39]; 1215 (Resveratrol) [40]	C–O phenolic; C–O–C ester
3013, 3014	3005, 3013	3013, 3016, 3019	2960, 2922 (Curcumin) [39]	C–H aliphatic (CH_2_, CH_3_ asymmetric stretching)
3740, 3798–3802	3742, 3794, 3793, 3797, 3800	3797	3265 (Curcumin) [39]; 3174–3248 (Resveratrol) [40]	O–H phenolic stretching (hydrogen bonded; broad)

* The first values separated by semicolons in the first three columns belong to curcumin in the hybrid molecule, while the others correspond to resveratrol.

### 3.2. Molecular Docking and Interaction Analysis

In our study, the binding scores (kcal/mol) of curcumin, resveratrol, their analogues, three designed hybrid molecules, and the reference inhibitor inavolisib on three different target proteins—AKT1, MAPK, and STAT3—are presented in Table 3 using the molecular docking technique. The docking scores indicate that the hybrid molecules exhibit stronger binding affinities to the target receptors compared to curcumin and resveratrol. Similarly, the hybrid molecules exhibit a competitive profile compared to their analogues. It is particularly noteworthy that the ELRC-LC hybrid shows a binding score of −11.4 kcal/mol on the AKT1 receptor, while curcumin and resveratrol exhibit binding scores of −9.8 kcal/mol and −8.3 kcal/mol, respectively. It is also remarkable that the synthetic inhibitor inavolisib, with a binding score of −9.9 kcal/mol, falls short of ELRC-LC. Furthermore, the ELRC-LC molecule demonstrates a binding score of −9.6 kcal/mol on the MAPK target protein, indicating that this hybrid provides the strongest interaction. Likewise, EtLRC shows strong affinity for AKT1 and MAPK, with binding scores of −10.8 and −9.3 kcal/mol, respectively, indicating stronger interactions of the hybrid molecules compared to pure curcumin and resveratrol. On the STAT3 target protein, the EtLRC (−7.6 kcal/mol) and ELRC-SC (−7.2 kcal/mol) hybrids display superior and equivalent binding values relative to the reference inhibitor (−7.2 kcal/mol). Overall, the hybrids appear to exert a more advantageous effect on receptors associated with the AKT1 and MAPK pathways, while maintaining a competitive profile on the STAT3 receptor. The docking scores further confirm that the hybrid molecules achieve lower binding energies and stronger affinities than curcumin and resveratrol alone on the target receptors [45]. This finding is attributed to the integration of pharmacophore regions of these natural polyphenols, which possess rich pharmacokinetic profiles, into a single scaffold with different variations [46]. Thus, the hybrid molecules form stable receptor–ligand complexes by establishing multiple bonding interactions within the active-site pockets of the target receptors [36]. Our findings support this notion, as demonstrated by the docking scores in Table 3. The hybrid molecules not only formed conventional hydrogen bonds with the active-site amino acid residues of the target receptors but also established additional interactions with residues such as Arg273 (Figure 3). These supplementary interactions increased the contact points of the hybrid molecules, thereby enhancing the stability of the receptor–ligand complexes [24,47].

A review of the literature reveals that polyphenol-based hybrids exhibit significantly stronger binding affinities and multi-targeted effects than naturally occurring compounds. In the study conducted by Moreno-Quintana et al., curcumin–resveratrol hybrids were synthesized [24]. In this study, in addition to the in vitro methodology, molecular docking analyses were performed with AutoDock Vina. The hybrids were found to have binding scores of approximately −10.4, −8.5, −7.8, and −7.3 kcal/mol to the target receptors MMP-7, caspase-3, paspase-7, and p53, respectively [24]. These results demonstrate that the hybrids possess highly potent binding profiles across multiple targets. A recent study published in Scientific Reports showed that curcumin binds to the Transforming Growth Factor Beta 3 (TGFβ3) receptor through hydrogen bonding and π interactions, with a binding energy of −6.3 kcal/mol [48]. In this study, it was reported that polyphenols such as curcumin can establish stable contact with target receptors at multiple points within the active binding pockets. Our findings demonstrated that the ELRC-LC, ELRC-SC, and EtLRC hybrids, particularly on the AKT1 receptor, interacted with amino acid residues Trp80, Thr211, Leu264, and Lys268, and on MAPK with Tyr32, Glu33, Tyr36, Asp111, and Asp187, which are also common to the reference inhibitor inavolisib. Furthermore, additional interactions with residues Arg273 and Asp292 (AKT1) and Arg191 and Arg194 (MAPK), which enhance the stability and binding score of the receptor–ligand complex, provide a mechanistic explanation for the increased binding affinity, consistent with the literature [49]. The hybrid molecules designed in our study stand out with their significantly superior performance on AKT1 and MAPK compared to synthetic ligands, as well as their broad interaction repertoire. These hybrids exhibit a competitive profile on the STAT3 receptor, making them promising candidates for strategies aimed at the simultaneous suppression of the AKT1 and MAPK pathways, particularly in cancer treatment. Because our study is based on in silico analysis, our findings should be considered preliminary and hypothesis-generating. Nevertheless, the results concerning our hybrid molecules, which have not been previously reported in the literature, are presented as a guide for researchers aiming to confirm and further develop them through experimental studies.

**Table 3:** Comparative binding affinities (kcal/mol) of curcumin and resveratrol analogues designed hybrid molecules, and the synthetic inhibitor determined for AKT1, MAPK, and STAT3 receptors.

		Receptors		
	Ligands	AKT1	MAPK	STAT3
Resveratrol analogues	Resveratrol	−8.3	−7.6	−6.0
	ZINC000000006787	−8.3	−7.5	−5.7
	ZINC000003978779	−8.3	−7.2	−5.4
	ZINC000004098633	−8.2	−8.2	−6.9
	ZINC000015112534	−9.6	−7.6	−7.2
	ZINC000015112536	−9.7	−8.3	−6.7
	ZINC000015112538	−9.8	−8.1	−6.7
	ZINC000015112540	−9.8	−7.1	−6.3
	ZINC000035653092	−9.7	−7.4	−6.4
	ZINC000040977346	−9.0	−7.4	−5.5
	ZINC000085612047	−8.1	−9.5	−6.8
	ZINC000095620822	−10.6	−7.3	−6.7
	ZINC000100823225	−8.8	−10.3	−7.9
	ZINC000100823227	−11	−9.1	−6.8
	ZINC000100823228	−11.2	−9.3	−8.0
	ZINC000100827960	−11.3	−8.8	−6.9
	ZINC000100827962	−11.0	−8.8	−8.1
	ZINC000100827965	−11.0	−9.0	−8.2
	ZINC000230079510	−10.9	−9.9	−7.5
	ZINC000230079516	−11.8	−10.3	−8.3
	ZINC000230079520	−10.6	−10.2	−8.0
	ZINC000230079525	−10.8	−10.2	−7.5
	ZINC000230097101	−11.3	−9.2	−7.2
	ZINC000230097106	−10.8	−8.8	−7.7
	ZINC000230097112	−10.8	−8.5	−7.4
	ZINC000230097119	−11.0	−9.0	−7.4
Curcumin analogues	Curcumin	−9.8	−6.6	−5.6
	ZINC000000899824	−11.0	−7.4	−5.6
	ZINC000014948330	−9.1	−7.0	−5.3
	ZINC000016527488	−9.3	−6.8	−5.4
	ZINC000019816066	−9.7	−7.7	−6.4
	ZINC000085926636	−9.0	−6.4	−5.2
	ZINC000150366575	−10.8	−7.4	−6.9
	ZINC000150366578	−10.3	−7.7	−6.6
	ZINC000150366582	−10.2	−6.9	−6.6
	ZINC000150366588	−10.8	−7.1	−6.8
	ZINC000150368101	−10.2	−8.3	−7.1
	ZINC000150368109	−11.0	−9.0	−7.4
	ZINC000150368115	−10.8	−8.9	−6.9
	ZINC000150368122	−9.8	−9.3	−8.1
	ZINC000150368128	−10.2	−7.1	−6.1
	ZINC000150368132	−9.7	−7.6	−6.1
	ZINC000150368137	−10.4	−8.1	−7.0
	ZINC000150368142	−8.8	−7.7	−7.4
Hybrid Molecules of Curcumin-Resveratrol	ELRC-LC	−11.4	−9.6	−5.7
	ELRC-SC	−9.5	−7.8	−7.2
	EtLRC	−10.8	−9.3	−7.6
	Inavolisib	−9.9	−8.1	−7.2

### 3.3. MD Simulation Analysis

#### 3.3.1. Conformational Flexibility Analysis

The 100 ns MD trajectories were analyzed to investigate the conformational changes in the interactions of curcumin, resveratrol, the ELRC-LC, ELRC-SC, and EtLRC hybrid molecules, as well as the synthetic inhibitor inavolisib, with the AKT1, MAPK, and STAT3 receptors. The binding affinities of these ligands to the active sites of the target proteins were assessed through Root Mean Square Deviation (RMSD) analysis, and the corresponding results are presented in Figure 5A1–A3. Curcumin and the hybrid molecules appear to equilibrate rapidly in ligand complexes formed with the AKT1 receptor (Figure 4A1). It is particularly noteworthy that the EtLRC hybrid maintained low RMSD values of approximately 0.3 nm, similar to the reference inhibitor, throughout the simulation. This finding indicates the persistence of π–π interactions with Trp80, π-cation interactions with Arg273, and hydrophobic contacts mediated by Ile84 and Lys268 in the binding interaction maps of the EtLRC molecule analyzed both after docking and after MD simulation [50]. The fluctuations observed in the resveratrol complex throughout the simulation indicate less stable binding, which can be attributed to the transient loss of critical interaction contacts [20,36]. MAPK complexes (Figure 4A2) exhibited higher RMSD values and greater fluctuations compared to the AKT1 receptor complexes, particularly for curcumin and inavolisib. Curcumin and inavolisib reached equilibrium at approximately 3.0 nm and 2.2 nm after 74 ns and 53 ns, respectively. The pronounced fluctuations observed with curcumin, especially after 50 ns, indicate partial unwinding of the α-helical regions [51]. Resveratrol, a natural polyphenol, exhibited a stable structure around 0.3 nm compared to curcumin. However, the slightly increasing plateau observed after 77 ns suggests that the ligand did not fully occupy the critical hydrophobic subpockets and was adopting a new conformation [52]. In the MAPK receptor complexes, the hybrid molecules maintained a more stable range between 0 and 1 nm throughout the simulation, indicating a generally higher stability compared to the natural polyphenol alone [24]. The continuous plateau of the ELRC-LC hybrid around 0.8 nm indicates that this molecule represents the most stable hybrid. This stability is primarily attributed to the long aliphatic chain embedding into hydrophobic amino acid subpockets, such as Val39 and Ala35, thereby establishing van der Waals and π-alkyl interactions [53]. Moreover, the preservation of π–π stacking between the aromatic nucleus and Tyr113 prevents water entry into the long-chain pocket, thereby reducing the desolvation penalty, maintaining low RMSD values, and supporting the formation of a stable complex structure [54]. Similarly, ELRC-SC, which forms a stable complex structure, cannot access the second hydrophobic subpocket due to its shorter aliphatic chain. However, it exhibits a stable plateau around 1.0 nm through conventional hydrogen bonds with amino acid residues such as Gly32, Val39, Met108, Asp111, and Asn154. Since this hybrid establishes relatively fewer hydrophobic anchoring points than ELRC-LC, an increase in RMSD values is observed [55]. EtLRC, designed as the final hybrid molecule, exhibits a stable trajectory particularly between 47 and 80 ns. However, significant fluctuations, reaching up to 1.5 nm after 80 ns, can be attributed to the flexibility and polarity of the ether bridge within the hybrid structure [56]. The increased hydrogen bond acceptor capacity of the ether bridge causes the ligand in the binding pocket to rely on water-mediated hydrogen bonds and to interact with solvent molecules [57]. Since these hydrogen bonds are transient, the continuity of π-alkyl interactions with hydrophobic residues such as Val39, Ala52, Met108, Leu156, and Cys166 is disrupted [54]. Interruptions in the interactions increased the center-of-mass (COM) distance of the ligand from the receptor, causing the hybrid to undergo conformational shifts throughout the simulation [58]. In the reference inhibitor complex, inavolisib exhibited RMSD values hovering around 2.0 nm. The plateau, reaching equilibrium at approximately 2.2 nm after 52 ns, indicates that inavolisib adopted a binding pocket conformation that is correct but comparatively looser than those of the hybrid molecules.

In the STAT3 receptor complexes (Figure 4C1), the curcumin–resveratrol hybrids formed multiple interactions within the active pocket of the receptor, further emphasizing their stability. Among these hybrids, conventional hydrogen bonds were observed with Glu324, Gln326, Gly254, Ser514, and Ser509; π-alkyl and hydrophobic contacts with Pro256, Ile258, Trp501, Ala505, Ile522, and Leu525; and a π-sulfur interaction with Cys251. These multiple interactions maintained complex stability throughout the simulation, keeping RMSD values below approximately 1.0 nm. Resveratrol exhibited a comparatively more stable structure on this receptor than the other ligands. However, the disappearance of hydrophobic interactions present before docking—particularly the hydrogen bonds with Arg350 and Ser540—at the end of the MD simulation indicates an increase in RMSD values in the hybrids [57].

The flexibility of amino acid residues in complexes formed by natural polyphenols, hybrid molecules, and the reference inhibitor with AKT1, MAPK, and STAT3 receptors was calculated using the backbone root mean square fluctuation (RMSF) method and is presented in Figure 4B1–B3. Generally, the peaks in the graphs of these complexes are located in the terminal and loop regions. Relatively lower flexibility is observed in the regions of the other amino acid residues [59]. In the hybrid complexes designed in our study, fluctuations were observed between 0.1 and 0.5 nm, and peak values were suppressed. This was particularly evident in regions adjacent to the binding pocket at Trp80 and Thr21 (Figure 4B1) for the AKT1 receptor, and Tyr36 and Asp111 for the MAPK receptor (Figure 4B2), suggesting the presence of persistent hydrogen bonds and π–π interactions. The high fluctuations in some loop regions of resveratrol on both receptors indicate poor stabilization due to hydrogen bond losses [60,61]. Curcumin, on the other hand, is less restrictive than the hybrid molecules. The reference inhibitory ligand exhibited local fluctuations on the receptors, indicating that it had reached the correct conformation at the correct site. However, the hybrid molecules provided much stronger local rigidity than the reference ligand. Our findings from the RMSF calculations are consistent with the RMSD data, highlighting that the hybrid molecules exhibit local rigidity in the receptor binding pockets, thereby increasing conformational and structural stability. In the STAT3 complexes, RMSF values averaged around 0.3 nm. The ELRC-LC and EtLRC hybrids exhibited relatively lower peak amplitudes than the other ligands, indicating that these structures provided significant local rigidity within the binding site [24]. Curcumin and resveratrol polyphenols exhibited high fluctuations and RMSF values in the loop regions of the target receptor, demonstrating the superiority of the hybrids in terms of stability, in parallel with the RMSD results. Our findings are consistent with literature studies targeting different proteins. For instance, in a study by Alkhathami et al., a 500 ns simulation of curcumin on TGFβ3 was performed [48]. In this study, although curcumin exhibited stable binding, the RMSF values revealed fluctuating stability. These findings are consistent with the RMSD values obtained for curcumin in our study. Similarly, a study on curcumin–resveratrol hybrids reported that the hybrid molecules exhibited much lower RMSD values and more stable binding to colorectal cancer cells compared to natural polyphenols alone [24]. In this study, the hybrids were observed to exhibit stable binding through multiple hydrophobic and π–π interactions. In summary, the findings from literature studies are consistent with the qualitative and quantitative results of our study, suggesting that hybrid molecules may be potential candidates for novel structures that provide superior stability compared to single molecules such as curcumin and resveratrol across multiple targets.

#### 3.3.2. Radius of Rotation (Rg)

To understand the structural compactness of the proteins, the radius of gyration (Rg) of the receptor–ligand complexes is shown in Figure 5C1–C3. During the MD simulations, all ligand complexes of the AKT1 receptor oscillated around 2.25 nm (Figure 4C1). The ELRC-LC hybrid molecule formed a plateau with low-amplitude fluctuations at 2.2 nm, indicating that its long aliphatic chains localized to the hydrophobic cores and maintained compact protein integrity [62]. Despite having a short covalent chain, ELRC-SC maintained compactness through hydrogen bonding with polar amino acid residues [57]. Similarly, the stability of EtLRC in terms of compactness at a value of 2.15 nm was attributed to the formation of water-mediated bonds through its ether bridge, as well as multiple contacts with regions occupied by Leu and Ile residues in the aliphatic region [63]. The natural polyphenol curcumin also formed hydrogen bonds and hydrophobic anchors on the receptor through the phenolic –OH groups and aromatic rings in its structure [10]. However, the flexible structure of this molecule caused greater fluctuations compared to the other complexes. The resveratrol complex, on the other hand, has a smaller chemical structure than the other ligands. Therefore, although it cannot reach many contact points, its ability to form stable hydrogen bonds through –OH groups contributes to its compactness [64]. The synthetic inhibitor inavolisib showed higher Rg values and fluctuations at 2.28 nm compared to both the native and hybrid molecules. The elevated Rg profile suggests a more flexible binding mode, indicating relaxation of the AKT1 receptor pocket [65]. In complexes with the MAPK receptor, oscillations around 3.5 nm were observed due to the receptor’s bulkier structure (Figure 4C2). Among the hybrids, EtLRC exhibited the narrowest fluctuation range, around 3.2 nm, with the MAPK receptor. It is suggested that the hydrophobic tails in this structure increase compactness by forming multiple contacts with hydrophobic amino acid residues in the MAPK receptor [66]. ELRC-LC, on the other hand, maintained its compact complex structure by conforming to the binding surface of the receptor with its long covalent chain. ELRC-SC, in contrast, exhibited a higher wavy plateau at 3.45 nm compared to the other hybrids. Its short covalent chain structure obstructed access to the second hydrophobic pocket of the receptor, suggesting weaker compactness compared to the other ligands [67]. Natural polyphenols, on the other hand, exhibited higher Rg values around 3.4 nm, but overall compactness was still evident. Inavolisib, similar to its behavior with AKT1, showed a more highly wavy profile, while the hybrid molecules appeared to adopt a much more compact conformation on the receptors than the reference inhibitor. For the STAT3 receptor, Rg values remained similarly stable between 3.2 and 3.5 nm. Among the hybrid molecules, ELRC-LC and EtLRC exhibited fewer fluctuations, particularly in the target receptor, thereby strengthening receptor integrity and preserving the compact structure. Natural polyphenols exhibited larger fluctuations over time compared to the hybrids, but generally maintained the compact complex structure [68]. In general, the designed hybrid molecules appeared to increase the compactness of protein structures. This effect is primarily attributed to the hybrids’ ability to simultaneously optimize hydrogen bonding and hydrophobic interactions [54]. A review of literature studies reveals similar trends regarding the Rg parameter. In a study by Asl et al., curcumin was reported to bind to the pro-apoptotic Bak and Bad proteins [69]. Although Rg values increased due to protein relaxation upon binding, compactness in the Bim protein was reported to persist. This suggests that the flexible structure of curcumin may negatively affect compactness in certain proteins [69]. In our study, the long and short aliphatic chains and ether bridges in the structures of the designed hybrid molecules were found to increase compactness by providing multiple anchoring sites within the hydrophobic pockets of the receptors compared to natural polyphenols. A similar observation was reported for the resveratrol polyphenol in a literature study by Novak et al. [70]. Resveratrol binding to the 11β-HSD-1 receptor remained constant with average Rg values of approximately 17.8 ± 0.3 Å, demonstrating that the compactness of the complex was maintained. While this finding from the literature is significant, it is evident that the compactness of resveratrol is not enhanced due to structural limitations. Our study, however, concluded that compared to individual molecules, the chemical structures and pharmacological profiles of the hybrid molecules allow for both increased and sustained compactness. In the study conducted by Moreno-Q et al. on curcumin–resveratrol hybrids, it was found that the hybrid molecules tended to increase compactness by forming multiple interactions with different receptors [24]. These literature data support the formation of functional and stable complexes of the hybrids on receptors and the increase in structural compactness, thereby paralleling our findings.

#### 3.3.3. Binding Free Energy MM/PBSA Analysis

Table 4 shows the basic components of the binding energies of receptor–ligand complexes obtained from MM/PBSA free energy decomposition. A comparison of the initial (0 ns) and final (100 ns) MD simulation values reveals the conformational adaptation of all ligands, especially hybrid ligands, in the active site of the receptor. The ΔG_bind_ values of the natural polyphenol curcumin on AKT1, MAPK, and STAT3 receptors in the last frame are −13.38, −32.28, and 17.60 kcal/mol, respectively. Curcumin is a diferuloylmethane derivative formed by the linkage of two phenolic rings through a β-diketone bridge [71]. Phenolic hydroxyl groups, with their π-conjugation and flexible central linker, facilitate the formation of hydrogen bonds as well as hydrophobic interactions [72]. However, the high conformational flexibility of the β-diketone group in its structure limits its ability to form stable anchorage within the cavities of AKT1 compared to other receptors [73]. Our findings suggest that hydrogen bonding occurs during docking with Gln105, but stable hydrogen bonds are formed with ASP167 and LYS54 during MD simulation [74]. In particular, the AKT1–curcumin complex exhibits a relatively constant shift of +2.27 kcal/mol, whereas the MAPK receptor shows a shift of −14.58 kcal/mol, indicating better localization within the receptor’s active pocket. This finding is consistent with the interpretations of Rout et al., who reported that polyphenols such as demethoxycurcumin and curcumin exhibit high-affinity binding with free binding energies ranging from −64.86 to −48.53 kcal/mol [75].

Resveratrol exhibits relatively higher binding energies with curcumin complexes (AKT1: −12.46 kcal/mol; MAPK: −17.20 kcal/mol; STAT3: −20.57 kcal/mol), which is attributed to the limited aromatic surface area in its structure, thereby reducing van der Waals contributions [54]. In the study by Akash et al., which reported the free binding energies of resveratrol derivatives on the APC protein, a value of −1.11 kJ/mol was observed for the resveratrol 3-β-mono-D-glucoside ligand [76]. The fact that the values for our hybrid molecules are even better than this finding is crucial for ensuring high conformational stability. The hybrids designed in our study exhibited significant superiority over other ligands on each receptor, highlighting the rational contribution of skeletal modification to their binding free energies [77]. The high van der Waals contribution (−75.42 kcal/mol) and sustained electrostatic interactions (−29.64 kcal/mol) of the EtLRC hybrid in the AKT1 receptor resulted in the strongest binding (ΔG_bind_ = −26.50 kcal/mol). The decrease in this ligand to −25.19 kcal/mol at the end of the simulation indicates that the hybrid’s conformational flexibility enables proper positioning within the binding pocket, resulting in deep hydrophobic packing [78]. The ELRC-LC and ELRC-SC hybrids reached binding energy values of −22.11 and −22.95 kcal/mol, respectively, at the end of the simulation, indicating the formation of strong complexes. Notably, the reference inhibitor complex, inavolisib, with a binding energy of −16.06 kcal/mol, showed weaker affinity, indicating that the hybrid molecules possess a binding profile comparable to both natural polyphenols and clinical-grade synthetic inhibitors. The hybrid molecules also exhibited strong interactions with other receptors at the end of the simulation. The strongest interaction was observed with the MAPK receptor, where the initial positive binding energy of ELRC-SC (+1.77 kcal/mol) decreased to −36.29 kcal/mol. This demonstrates that the hybrid structure forms a stable complex through electrostatic and van der Waals contributions during the conformational process within the ATP-binding pocket [54,79]. Likewise, hybrid molecules exhibited strong binding in STAT3 complexes. EtLRC, in particular, showed a binding energy of −35.40 kcal/mol on this receptor, indicating that the hybrid fits well into the dimerization region due to the flexibility provided by the ether bridge [80]. While the free binding energies of the initial frames in hybrid molecule complexes generally indicate an unstable complex structure, the values that change positively at the end of the simulation show that the interactions between the receptors and the hybrid molecules improve and strengthen over time [81]. This finding highlights not only the high binding affinities of the hybrids but also their dynamic binding stability.

**Table 4:** Binding Free Energy Calculations Using MM-PBSA for Receptors–Ligand Interactions (kcal/mol).

Receptors	Ligands	VDWAALS		EEL		Total (ΔG_bind_)	
Frame Types		Initial	Last	Initial	Last	Initial	Last
AKT1	Curcumin	−44.97	−46.02	−28.71	−0.70	−15.65	−13.38
	Resveratrol	−28.99	−27.56	−11.45	−16.70	−4.54	−12.46
	ELRC-LC	−70.94	−69.99	−34.00	−30.53	7.91	−22.11
	ELRC-SC	−56.97	−44.73	−28.13	−71.10	1.63	−22.95
	EtLRC	−72.42	−75.42	−6.32	−29.64	1.31	−26.50
	Inavolisib	−47.83	−26.48	−63.91	−71.86	7.16	−16.06
MAPK	Curcumin	−39.67	−41.70	−43.20	−39.95	−17.70	−32.28
	Resveratrol	−26.20	−25.51	−31.47	−37.46	−12.66	−17.20
	ELRC-LC	−50.37	−38.24	−45.44	−50.66	−12.51	−26.21
	ELRC-SC	−66.23	−47.65	−29.87	−52.83	1.77	−36.29
	EtLRC	−57.92	−28.68	−17.8	−52.03	−10.98	−29.38
	Inavolisib	−40.62	−19.36	41.74	−65.61	37.29	−4.63
STAT3	Curcumin	−36.94	−32.55	−7.35	2.84	−15.80	−17.60
	Resveratrol	−22	−20.39	−24.65	−40.82	−11.64	−20.57
	ELRC-LC	−43.67	−37.52	−18.09	−26.89	−7.53	−14.94
	ELRC-SC	−42.89	−19.89	−22.80	−18.35	−15.41	−17.83
	EtLRC	−51.74	−61.97	−17.75	−21.62	−22.25	−35.40
	Inavolisib	−29.94	−26.60	−55.96	−114.74	−6.68	−29.14

## 4. Materials and Methods

### 4.1. De Novo Hybrid Drug Design

In our study, three curcumin–resveratrol hybrid molecules were designed using Avogadro (v1.2.0) software to improve the bioavailability and pharmacokinetic profiles of curcumin and resveratrol individually. Based on the literature, two ester-linked hybrid molecules—a long-chain ester-linked curcumin–resveratrol hybrid (ELRC-LC) and a short-chain ester-linked curcumin–resveratrol hybrid (ELRC-SC)—were designed to evaluate the effect of alkyl chain length in curcumin and resveratrol on receptors, particularly in cancer cell signaling pathways. Additionally, an ether-linked curcumin–resveratrol hybrid (EtLRC), characterized by a distinct bond type, was designed in our study. The resulting hybrid molecules were subjected to energy minimization using the MMFF94 force field. The design strategy for these hybrid molecules, in conjunction with previous studies, consists of covalently attaching the phenolic hydroxyl groups of resveratrol to the methoxyphenolic or β-diketone regions of curcumin using Avogadro (v1.2.0) in silico. In the ELRC-LC and ELRC-SC hybrid molecules, the esterification mechanism was modeled on a nucleophilic acyl substitution strategy [82]. In this strategy, we used activated carboxylic acid derivatives derived from the curcumin binding site as electrophiles, and the phenolic hydroxyl group of resveratrol as nucleophiles. In this context, two different types of ester-linked hybrid molecules were formed: long and short chains [36]. Finally, in our study, the ether-linked hybrid molecule (EtLRC) was designed according to the principles of Williamson ether synthesis [83]. A covalent ether bridge was formed between curcumin and resveratrol through the alkylation mechanism of the phenolic oxygen atom of resveratrol with an electrophilic spacer linked to the phenolic hydroxyl group of curcumin [64,83]. Ether bridges exhibit important properties due to their higher conformational flexibility, their ability to form solvent-mediated hydrogen bond interactions as a result of their strong electron-donating character, and their contribution to increasing the overall polarity of the molecule [32]. Owing to these properties, EtLRC exhibits a different structural profile compared to ester-linked hybrid molecules and was designed to comparatively analyze the pharmacochemical properties of ether and ester linkages within our design strategy. In the designed EtLRC hybrid molecule, the main bridge between curcumin and resveratrol is formed by an ether bond, while the side chains contain ester functional groups. Although the hybrid molecule contains both ester and ether bonds, the nomenclature of all molecules was based on the bond type of the main bridge. Three-dimensional representations of the structures designed in this study are presented in Figure 5.

### 4.2. Geometry Optimization Using Density Functional Theory (DFT)

Geometry optimization was performed at the density functional theory (DFT) level to obtain the most stable conformations of the energy-minimized resveratrol–curcumin hybrid molecules (ELRC-LC, ELRC-SC, and EtLRC), which have not been directly reported in the literature before. TURBOMOLE V7.5 software, employing the def2 basis set and capable of performing high-accuracy calculations on complex molecules, was used for this process. The def2-TZVP (triple-zeta valence with polarization) basis set, recognized for its high computational accuracy, together with the B3LYP functional, widely applied to estimate electron density and molecular orbital distribution, was employed in the optimization process. All these steps were performed to produce physically reliable and reproducible results for these previously unreported ligands. The optimized and stable hybrid ligands obtained were evaluated as reference geometries and subsequently prepared for the molecular docking stage [84]. Subsequently, for the theoretical spectroscopic characterization of these hybrid structures, FTIR spectra were computed at the B3LYP/def2-TZVP level, and the functional groups of the molecular structures were compared with the literature. The electronic properties of the optimized crystal structures were visualized using the Highest Occupied Molecular Orbital (HOMO) and Lowest Unoccupied Molecular Orbital (LUMO) distributions, as well as molecular electrostatic potential (MEP) maps. In summary, the crystallographic, structural, and electronic properties of the hybrid molecules designed in our study were revealed with high reliability and accuracy using in silico techniques [85].

### 4.3. Molecular Docking

Curcumin, resveratrol, their analogs obtained from the ZINC database “https://zinc.docking.org/ (accessed on 27 June 2025)”, and hybrid derivatives drawn within the scope of our study were analyzed at the molecular level for their interaction potential on target proteins involved in oncogenic signaling pathways. AKT1 (PDB ID: 8UW9), MAPK (PDB ID: 8ZJV), and STAT3 (PDB ID: 6NUQ) proteins, which are central to signaling pathways regulating cell proliferation, metastasis, and apoptosis, were selected as target receptors. These receptors were chosen because they are central regulators of the PI3K/AKT, MAPK, and JAK/STAT pathways, the dysregulation of which has been linked to breast cancer. These protein structures are clinically and translationally important because they have the potential to increase apoptosis and suppress metastatic progression. At this stage, the synthetic inhibitor inavolisib, which plays an important role in suppressing the PI3K/Akt/mTOR pathway and has proven clinical efficacy, was chosen as the reference ligand for all target protein structures [86]. Thus, the binding interactions and mechanisms of all ligand structures used in our study were presented in comparison with the synthetic ligand with high clinical success potential.

The three-dimensional crystal structures of the proteins in question were obtained from the Protein Data Bank database “https://www.rcsb.org/ (accessed on 27 June 2025)” These receptor structures were individually opened using USCF Chimera 1.17.3 software to purify the co-crystallized ligand, water molecules, and ion structures. Polar hydrogen atoms and Gasteiger charges were then assigned and saved in the appropriate format [73,87].

Each prepared receptor structure was loaded into AutoDockFR (ADFR) to determine the grid box center coordinates. Grid box parameters were defined by selecting amino acid residues from the active and binding sites reported in the literature [73,88]. The defined grid box center coordinates of the receptors and other relevant parameters are presented in Table 5.

To prepare the ligands for molecular docking, curcumin, resveratrol, and their analogs retrieved from the ZINC database were individually loaded into UCSF Chimera 1.17.3, and polar hydrogens were added. They were then converted to the appropriate format by defining torsional degrees of freedom and rotation centers [87,89]. Similarly, the same procedures were applied to the stable hybrid structures derived from the structural combination of curcumin and resveratrol, first proposed in this study.

After completing all docking preparations, molecular docking analyses were performed using AutoDock Vina (version 1.1.2). The exhaustiveness parameter was set to 10, and nine distinct binding conformations were generated for each ligand. Among these, the conformation exhibiting the lowest binding free energy (ΔG_bind_, kcal/mol) was selected as the most favorable receptor–ligand complex for subsequent analyses [73]. The binding interaction profiles, including hydrogen bonds, hydrophobic interactions, and π–π stacking interactions, were further examined and visualized using Discovery Studio Visualizer 2021. Validation of the docking step was achieved by docking the clinically approved synthetic inhibitor inavolisib with AKT1, MAPK, and STAT3 receptors. This inhibitor was selected as a positive control due to its robust performance in inhibiting the PI3K/AKT/mTOR pathway. The reliability of the protocol was confirmed by selecting complexes with an RMSD of less than 2.0 Å and by comparing each docking score with the reference configuration.

### 4.4. Molecular Dynamics Simulation

To investigate the conformational stability and flexibility of the best candidate receptor–ligand complexes obtained from docking, 100 ns molecular dynamics (MD) simulations were carried out using the high-performance computing environment GROMACS 2019.1. Each receptor–ligand complex was separated to generate coordinate and topology files. The coordinate and topology files of the receptors were prepared in the GROMACS environment using the CHARMM36-jul2022 force field, whereas the ligand topology and coordinate files were generated via the SwissParam web server. The receptor and ligand files prepared for the simulations were then merged in a GROMACS-compatible format, followed by solvation, energy minimization, and equilibration procedures. The prepared systems of the complexes were solvated in a triclinic water box using the TIP3P explicit water model. The distance between the box edges and the solute was set to 1.0–2.0 nm depending on the structure of each complex. Na^+^ and Cl^−^ ions were added at a 0.1 M concentration to neutralize the system. Energy minimization was performed using the steepest descent algorithm. Subsequently, NVT equilibration (100 ps, 300 K, V-rescale thermostat) and NPT equilibration (100 ps, 1 bar, Parrinello–Rahman barostat) were carried out, while restraining the positions of the heavy atoms [90,91]. Long-range electrostatic interactions were calculated using the Particle Mesh Ewald (PME) method, while short-range van der Waals and Coulomb interactions were treated with cutoff distances of 1.0 nm. A 100 ns production run with a time step of 2 fs was then carried out for each prepared complex [35]. At the end of the simulation, RMSD, RMSF, and Rg analyses were performed using GROMACS and visualized with the QtGrace program.

### 4.5. MM/PBSA-Based Binding Free Energy Calculations

Binding free energies of the receptor–ligand complexes were calculated using the gmx_MMPBSA package integrated with GROMACS. The molecular mechanics/Poisson–Boltzmann surface area (MM/PBSA) method was employed for these calculations. The CHARMM36-jul2022 force field was again applied to determine the binding energies [90]. Calculations for the complexes were performed using the initial and final frames of the trajectories obtained from the 100 ns MD simulations. The changes in binding free energy throughout the simulation are presented in a comparative manner. The binding free energies were calculated according to Equations (1) and (2) [35]. In Equation (2), ${\Delta G}_{MM}$ represents the molecular mechanical interaction energy, corresponding to the sum of the van der Waals and electrostatic components. ${\Delta G}_{PB}$ denotes the polar solvation free energy, while ${\Delta G}_{SA}$ denotes the nonpolar solvation free energy. However, due to the high computational cost, the −TΔS (entropy) term was not included in the calculations. In this study, the total sum of all energy components was considered as the basis for the binding free energy analyses [35].

(1) $${\Delta G}_{bind}=G_{complex}-(G_{protein}+G_{ligand})$$

(2) $$\Delta G={\Delta G}_{MM}+{\Delta G}_{PB}+{\Delta G}_{SA-T\Delta S}$$

## 5. Conclusions

In our study, ELRC-LC, ELRC-SC, and EtLRC hybrid molecules, rationally designed de novo through the combination of curcumin and resveratrol, were comprehensively evaluated through DFT-based Highest Occupied Molecular Orbital (HOMO)–Lowest Unoccupied Molecular Orbital (LUMO), Molecular Electrostatic Potential (MEP), and Fourier Transform Infrared (FTIR) analyses, molecular docking, molecular dynamics simulations, and binding free energy calculations. The finding that the hybrid structures preserved the essential functional groups of curcumin and resveratrol was confirmed by FTIR and MEP mapping, thereby ensuring electronic stability. Molecular docking results revealed that the hybrid molecules exhibited stronger binding affinities than natural polyphenols and even the reference inhibitor Inavolisib. The strong binding of the ELRC-LC hybrid to AKT1 and MAPK receptors was particularly noteworthy. Molecular dynamics simulations confirmed the highly stable conformations of the hybrids within the receptor binding pockets, with low RMSD values confirming the continuity of hydrophobic interactions and hydrogen bonds throughout the simulation. Binding free energy calculations using the MM/PBSA method revealed that the hybrids formed more stable complexes with receptors compared to other ligands. The findings demonstrate that hybrid compounds derived from curcumin and resveratrol can overcome pharmacokinetic limitations compared to natural polyphenols alone, while simultaneously suppressing the AKT1, MAPK, and STAT3 signaling pathways, which play crucial roles in apoptosis resistance, proliferation, and metastasis of cancer cells, thereby representing promising multi-target anticancer agents. While the study presents promising findings, further in vivo or in vitro experiments are necessary to confirm the anticancer activities of these proposed potent cancer pathway modulators. In summary, our study demonstrates the potential of rationally designed hybrid molecules to act as de novo hybrid inhibitors through in silico analysis. However, this study serves as a reference point for the investigation of these hybrid molecules in in vitro and in vivo studies and can guide experimentally based research for the optimization and validation of these curcumin–resveratrol-derived hybrids.

## Figures and Tables

**Table 1:** Electronic structure and reactivity descriptors of hybrid molecules based on HOMO–LUMO analysis (eV).

Hybrid Molecules	HOMO	LUMO	GAP	χ	η	σ	μ	ω
ELRC-LC	−5.5786	−2.3468	3.2318	3.9627	1.6159	0.6189	−3.9627	4.8589
ELRC-SC	−5.6607	−2.0453	3.6154	3.8530	1.8077	0.5532	−3.8530	4.1062
EtLRC	−5.1430	−2.3158	2.8272	3.7294	1.4136	0.7074	−3.7294	4.9195

**Table 5:** Structural Preparation Parameters and Active-Site Residues of Target Proteins Employed in Molecular Docking Studies.

Receptors	PDB ID	Grid Center Coordinates (Å)	Grid Box Dimensions (Å) *	Key Binding Site Residues
AKT1	8UW9	X: 10.950, Y: 10.531, Z: −32.793	30 × 30 × 30	Thr211
MAPK1	8ZJV	X: −26.719, Y: 17.156, Z: 4.932	30 × 30 × 30	Asp106, Met108, Asp111, Ser153, Asp167
STAT3	6NUQ	X: 10.076, Y: 51.043, Z: 5.082	30 × 30 × 30	Arg609, Ser611, Glu612, Ser613, Ser636, Glu638, Gln644, Tyr657

* Grid box dimensions were optimized according to the optimal binding conformations of the ligands with the target receptors. The amino acid residues of the active and binding sites were identified based on H-bond analyses from the PDBsum database (https://www.ebi.ac.uk/thornton-srv/databases/pdbsum/ (accessed on 3 July 2025)).

## Data Availability

Data is contained in the paper.

## Abbreviations

The following abbreviations are used in this manuscript:

RMSD	Root Mean Square Deviation
AKT1	AKT serine/threonine kinase 1
MAPK	Mitogen-Activated Protein Kinases
STAT3	Signal Transducer and Activator of Transcription 3
RMSF	Root Mean Square Fluctuation
Rg	Radius of Gyration
MM/PBSA	Molecular Mechanics Poisson-Boltzmann Surface Area
ELRC-SC	Ester-Linked Resveratrol–Curcumin Short-Chain
ELRC-LC	Ester-Linked Resveratrol–Curcumin Long-Chain
EtRC	Ester-Linked Resveratrol–Curcumin
DFT	Density Functional Theory
MD	Molecular Dynamics
NF-κB	Nuclear Factor kappa B
EGFR	Epidermal Growth Factor Receptor
ADMET	Absorption, Distribution, Metabolism, Excretion, and Toxicity
TGFβ3	Tumor Protein p53, Transforming Growth Factor Beta 3
